# StrongNet: An International Network to Improve Diagnostics and Access to Treatment for Strongyloidiasis Control

**DOI:** 10.1371/journal.pntd.0004898

**Published:** 2016-09-08

**Authors:** Marco Albonico, Sören L. Becker, Peter Odermatt, Andrea Angheben, Mariella Anselmi, Arancha Amor, Beatrice Barda, Dora Buonfrate, Philip Cooper, Laurent Gétaz, Jennifer Keiser, Virak Khieu, Antonio Montresor, José Muñoz, Ana Requena-Méndez, Lorenzo Savioli, Richard Speare, Peter Steinmann, Lisette van Lieshout, Jürg Utzinger, Zeno Bisoffi, StrongNet Working Group

**Affiliations:** 1 Centre for Tropical Diseases, Sacro Cuore Hospital, Negrar, Verona, Italy; 2 University of Torino, Torino, Italy; 3 Swiss Tropical and Public Health Institute, Basel, Switzerland; 4 University of Basel, Basel, Switzerland; 5 Institute of Medical Microbiology and Hygiene, Saarland University, Homburg/Saar, Germany; 6 Centro de Epidemiología Comunitaria y Medicina Tropical, Esmeraldas, Ecuador; 7 Mundo Sano Foundation, Buenos Aires, Argentina; 8 Facultad de Ciencias Medicas, de la Salud y la Vida, Universidad Internacional del Ecuador, Quito, Ecuador; 9 University Hospitals of Geneva and Faculty of Medicine, University of Geneva, Geneva, Switzerland; 10 National Center for Parasitology, Entomology and Malaria Control, Ministry of Health, Phnom Penh, Cambodia; 11 Department of Control of Neglected Tropical Diseases, World Health Organization, Geneva, Switzerland; 12 ISGlobal, Barcelona Centre for International Health Research, Hospital Clínic-Universitat de Barcelona, Barcelona, Spain; 13 Global Schistosomiasis Alliance, Chavannes de Bogis, Switzerland; 14 Tropical Health Solutions, Townsville, Queensland, Australia; 15 Division of Tropical Health & Medicine, James Cook University, Townsville, Queensland, Australia; 16 Department of Parasitology, Centre of Infectious Diseases, Leiden University Medical Center, Leiden, The Netherlands; Task Force for Global Health, UNITED STATES

## Introduction

Strongyloidiasis is a disease caused by an infection with a soil-transmitted helminth that affects, according to largely varying estimates, between 30 million and 370 million people worldwide [1,2]. Not officially listed as a neglected tropical disease (NTD), strongyloidiasis stands out as particularly overlooked [3]. Indeed, there is a paucity of research and public health efforts pertaining to strongyloidiasis. Hence, clinical, diagnostic, epidemiologic, treatment, and control aspects are not adequately addressed to allow for an effective management of the disease, both in clinical medicine and in public health programs [4]. The manifold signs and symptoms caused by *Strongyloides stercoralis* infection, coupled with the helminth’s unique potential to cause lifelong, persistent infection, make strongyloidiasis relevant beyond tropical and subtropical geographic regions, where, however, most of the disease burden is concentrated. Indeed, strongyloidiasis is acquired through contact with contaminated soil, and the infection is, thus, primarily transmitted in areas with poor sanitation, inadequate access to clean water, and lack of hygiene.

While the actual morbidity of chronically infected, immunocompetent individuals is subtle and difficult to appreciate [5], the particular importance of this parasitic worm is linked to its potential for maintaining lifelong autoinfections and causing a life-threatening hyperinfection syndrome in immunocompromised individuals [6]. Lack of point-of-care (POC) diagnostics and poor availability of, and access to, ivermectin (the current treatment of choice) are the two most significant bottlenecks that hinder effective management of the disease both in clinical and in public health settings. Examples of the management and importance of strongyloidiasis in two clinical contexts (in a tropical setting and a high-income country) and from a public health perspective are given in Boxes 1–3.

**Box 1. Individual Living in an Endemic Area with Diarrhea, Abdominal Pain, Pruritus, and Significant Dermatological Manifestations [10]**

A 43-year-old male farmer, living in the rural eastern part of Preah Vihear province, northern Cambodia, was diagnosed with a heavy *Strongyloides stercoralis* infection (924 and 478 larvae present in two Baermann examinations). Additionally, larvae and adult *S*. *stercoralis* were detected in Koga agar plate culture examinations of the stools. The patient was co-infected with hookworm and presented with abdominal pain, diarrhea, nausea, vomiting, fever, and a pronounced and persistent skin rash, which had been present with extensive itching for more than two years. The rash was observed on the back, chest, abdomen, and extremities and, due to frequent and intense scratching, showed signs of focal infection. Three weeks after treatment with a single oral dose of ivermectin (200 μg/kg) and a single oral dose of mebendazole, the patient’s rash had almost disappeared, and he was free of episodes of intensive itching.

**Box 2. In-Depth Diagnostic Assessment of Eosinophilia in High-Income Countries**

A 42-year-old, otherwise asymptomatic female patient from Croatia presented to a German hospital because of persistent peripheral blood eosinophilia of ≥15% and elevated IgE antibody titers. After an in-depth diagnostic assessment for hematologic and autoimmune disorders, several stool samples were analyzed for the presence of intestinal parasites. The formalin-ether concentration technique and the Baermann funnel technique were used, but neither helminth eggs nor intestinal protozoa cysts were identified. However, a serologic examination showed the presence of high anti-*Strongyloides* antibody titers. The patient was thus treated with oral ivermectin (200 μg/kg) for two days. After one week, the eosinophil counts were in the normal range, and repeated serology after three months showed a significant decrease of the anti-*Strongyloides* antibody titers. The patient had lived in Germany for more than a decade, but reported regular travels to rural Croatia. Even though Croatia is not considered to be an endemic country for human strongyloidiasis, this does not exclude that the patient acquired an infection with *S*. *stercoralis* there, as epidemiologic data from southeast Europe are scant and endemic areas may have remained undetected.

**Box 3. Impact of Preventive Chemotherapy with Ivermectin and Albendazole on the Prevalence of *S*. *stercoralis* and Other Infectious Agents [46,48,49]**

The Global Program to Eliminate Lymphatic Filariasis (GPELF) instituted annual administration of ivermectin and albendazole in Zanzibar (Pemba and Unguja Islands) from 2001 to 2006. After six rounds of community-based treatment with a coverage of more than 80%, lymphatic filariasis (LF) microfilaremia and antigen levels in sentinel and spot-check sites were below the thresholds of sustaining transmission, though a subsequent transmission assessment survey indicated that LF was not yet completely eliminated, especially from Pemba. Data from surveys prior to the GPELF showed prevalences of *S*. *stercoralis* of about 41% and 35% on Pemba and Unguja, respectively. After the termination of the GPELF intervention, the prevalence of *S*. *stercoralis* dropped on both islands to 7%. Despite the extremely high transmission of other helminths (i.e., *Ascaris lumbricoides*, *Trichuris trichiura*, and hookworm), a 90% decline in the health clinics records was shown. Moreover, scabies had been rampant on both islands, but the number of scabies cases that were reported from health services dramatically declined (by 68%–98%) in the years following the periodic treatment with ivermectin. Additionally, the combination of ivermectin with albendazole (or mebendazole) showed a higher efficacy than benzimidazole monotherapy against soil-transmitted helminth infections, particularly for *T*. *trichiura*. Ivermectin may have also contributed to the success of an ongoing malaria control intervention; indeed, malaria is now on the verge of elimination in Zanzibar, and it has been hypothesized that ivermectin-containing blood meals might have had an effect on malaria transmission through their negative impact on *Anopheles* mosquitoes.

In 2011, a web-based platform became operational with the aims of sharing information and supporting research collaborations on *S*. *stercoralis* (*Strongyloides* Sharing Platform; see http://ezcollab.who.int/ntd/strongyloidiasis). This platform was initiated by the Department of Control of Neglected Tropical Diseases, World Health Organization (WHO), with the support of the WHO Collaborating Centre on Strongyloidiasis in Negrar (Verona, Italy). The main research needs and areas of interest of the *Strongyloides* Sharing Platform are summarized in a Viewpoint published in *PLoS Neglected Tropical Diseases* in 2013 [1]. Three main areas of research were highlighted: (i) assessment of the prevalence and disease burden of strongyloidiasis in different epidemiologic settings; (ii) development of novel diagnostic methods, such as screening of patients at highest risk and clinical management approaches; and (iii) enhancing the availability of, and access to, ivermectin, including research and development of alternative drugs and treatment regimens.

In September 2015, some 40 scientists and public health experts—including representatives of leading institutes of tropical medicine, WHO, and WHO Collaborating Centers—met during the 9th European Congress on Tropical Medicine and International Health (ECTMIH) in Basel, Switzerland. The main objectives of the meeting were (i) to discuss recent progress with an emphasis on diagnostics and treatment of *S*. *stercoralis*, particularly since the establishment of the *Strongyloides* Sharing Platform; (ii) to provide an update on the global status of strongyloidiasis, particularly regarding the integration of *S*. *stercoralis* into the WHO’s preventive chemotherapy control strategy for soil-transmitted helminthiasis, and the steps required to achieve access to ivermectin treatment in endemic countries; and (iii) to outline a *Strongyloides*-related research agenda for the years to come. Moreover, the meeting provided an opportunity to launch a new research network–StrongNet. Of note, StrongNet is based on the existing *Strongyloides* Sharing Platform but has a more inclusive scope. It is thus open to any interested researcher and institution in order to foster collaborations and deepen exchange (e.g., establishment and exchange of stool and serum banks for *Strongyloides* research, multicenter drug and diagnostic studies, linking up researchers, public health experts, and connecting existing networks with related and overlapping interests).

During the meeting, the discussion addressed three main areas: (i) epidemiology and diagnosis of *S*. *stercoralis* infections; (ii) treatment options and global access to ivermectin; and (iii) public health strategies for the control of strongyloidiasis in endemic countries. Here, we review and summarize recent developments, describe the priorities identified during the September 2015 meeting, and outline specific recommendations and challenges for future research and public health interventions.

## Diagnosis of Strongyloidiasis

### Current Diagnostic Armamentarium and New Developments

Most *S*. *stercoralis* infections are chronic with an intermittent and low larval output. Hence, parasitologic methods, which visualize the presence of *S*. *stercoralis* larvae in stool, have only modest sensitivity [7–9], although the diagnosis is easy in heavy infections that are often found in highly endemic areas, as illustrated by the example in Box 1.

The Baermann funnel technique and the nutrient agar plate cultures (e.g., Koga agar plate) show reasonable sensitivity [11] with comparable diagnostic accuracy [12]. However, repeated stool sampling and a combination of both methods are recommended to achieve high sensitivity [10,13]. Importantly, though, the Baermann and Koga agar plate techniques are poorly standardized, which renders inter-laboratory comparisons difficult.

Peripheral blood eosinophilia is a useful, yet an unspecific, marker for infection with *S*. *stercoralis* in non-endemic areas (Box 2). Nonetheless, raised eosinophil counts may be absent, particularly (but not exclusively) in patients with a severe hyperinfection syndrome.

Because of their excellent sensitivity, serologic assays remain the mainstay of *S*. *stercoralis* screening, particularly in high-income countries, where serology can also be used to monitor treatment success. Antibody titers usually demonstrate sero-reversion or significantly decrease within months after successful treatment [14]. Moreover, serology lends itself to prevalence surveys in low- and middle-income countries (LMIC). It must be noted, though, that serology might give false-negative results in immunocompromised individuals and in recently acquired infections [15,16]. False-positive results are also possible in individuals with other parasitic infections; therefore, serologic testing must be interpreted with caution in areas where strongyloidiasis is endemic [17]. In a comparative assessment of serologic methods, two commercial and three in-house serologic tests showed reasonable sensitivity and a very high specificity over a given cutoff. Of note, a luciferase immunoprecipitation system-based assay (LIPS) that employs a recombinant antigen (NIE) was found to be the most specific method, but this assay is not widely available [9,18].

The Leiden University Medical Center (LUMC; Leiden, the Netherlands) developed and implemented a highly standardized multiplex real-time polymerase chain reaction (PCR) assay for a range of helminths, including *S*. *stercoralis* [19,20]. The technique showed high sensitivity according to a large number of samples subjected to this platform between 2006 and 2012, comprising patient samples from the Netherlands and several African, Asian, and South American countries.

Recent comparative studies performed in different settings evaluated conventional diagnostic methods and molecular tools for the detection of *S*. *stercoralis* in human stool samples and showed that quantitative real-time PCR is the most sensitive single stool-based diagnostic technique [21,22]. However, PCR may still miss cases detected by stool microscopy. Hence, a combination of real-time PCR with either Baermann or Koga agar plate is suggested to obtain high sensitivity. However, none of the fecal-based methods validated thus far has reached the sensitivity of serology.

In order to ease individual patient management and community-based prevalence studies, there is a need for a stool-based rapid diagnostic test (RDT) for *S*. *stercoralis* infection. Such a tool (i.e., a stool-based antigen detection test) is currently under development within the European Commission (EC)-funded National Infectious Disease Diagnostics (NIDIAG) research consortium (www.nidiag.org). A prerequisite for the development of such an RDT and to further improve existing diagnostic methods is the establishment of a “stool bank” in order to have access to well-characterized *Strongyloides*-positive stool samples, cryo-preserved live cultures, and/or frozen antigens. Such a “stool bank” will be facilitated through StrongNet. A bank of reference sera would be equally important. Serology could also be a useful complement for prevalence studies in endemic countries, especially if/when fully validated for use on dried blood spots collected on filter paper.

### Screening and Follow-Up of At-Risk Individuals

A targeted screening of individuals at risk of *S*. *stercoralis* is justified even in non-endemic areas by (i) the relatively high prevalence among migrants from endemic countries; (ii) the potential to diagnose autochthonous infections in some temperate countries that are considered non-endemic (e.g., parts of southern Europe); (iii) the availability of sensitive screening methods (e.g., serology and stool-based real-time PCR); (iv) an effective oral treatment with ivermectin; and, most importantly, (v) the opportunity to avoid the potentially fatal complications of disseminated strongyloidiasis. Expert consensus guidelines from the EC-funded research consortium “Coordinating Resources to Assess and Improve Health Status of Migrants from Latin America” (COHEMI; www.cohemi-project.eu) indicate that immunocompetent subjects with a high risk of exposure (e.g., immigrants from endemic areas, adopted children, and expatriates traveling abroad for more than one year) and immunocompromised individuals or patients who are likely to undergo immunosuppression (e.g., before organ transplantation), even if at low or intermediate risk of exposure to *S*. *stercoralis*, should be routinely screened for *S*. *stercoralis* infection with serology plus stool microscopy and/or real-time PCR. Furthermore, donor-derived transmission of strongyloidiasis during solid organ transplantation has been documented. Hence, specific guidelines should be employed for systematic screening of organ donors who might have been at risk of strongyloidiasis [23].

All positive patients should be treated with ivermectin. Furthermore, empiric treatment with ivermectin of patients at risk of immunosuppression is indicated without testing if past exposure cannot be excluded and in case of unavailable adequate diagnostic facilities. Post-treatment follow-up should be performed with the most sensitive technique available.

## Treatment of Strongyloidiasis: Access to Ivermectin Is a Bottleneck

Ivermectin is the current treatment of choice for strongyloidiasis [24–27]. Indeed, it is much more effective than other anthelmintic drugs such as albendazole, as has recently been confirmed by a Cochrane systematic review [28]. Ivermectin has improved the treatment of other human nematode infections and contributed to the successful control of a number of diseases, such as LF and onchocerciasis, some of which are today on the verge of elimination [29,30]. Half of the 2015 Nobel Prize in Medicine or Physiology was awarded to Drs. William C. Campbell and Satoshi Ōmura for the discovery of the nematocidal drug avermectin and its further development into ivermectin [31–33]. However, the optimal treatment regimen against strongyloidiasis remains to be determined. A randomized, multi-center trial is underway to determine the efficacy of single-dose ivermectin versus four doses for the treatment of uncomplicated strongyloidiasis. Participating study centers are located in Italy, Spain, and the United Kingdom (“StrongTreat 1 to 4;” ClinicalTrials.gov identifier: NCT01570504).

For severe cases in which oral treatment is not feasible, subcutaneous ivermectin (which is licensed for veterinary use only) has been employed in different treatment centers and warrants further clinical investigation [34]. In endemic settings, preventive chemotherapy with single-dose ivermectin in combination with albendazole may considerably impact infection rates with *S*. *stercoralis* and other infectious agents. However, the efficacy of this regimen and its long-term benefit on *S*. *stercoralis* morbidity in spite of possible re-infections remains to be determined. A major challenge identified by the Basel meeting participants is the highly restricted access to ivermectin in many LMIC where strongyloidiasis is most prevalent. For instance, there are countries where the drug is not available outside control programs for the elimination of LF and onchocerciasis. Moreover, in areas of sub-Saharan Africa where loiasis is endemic, ivermectin might cause severe adverse events (i.e., encephalopathy) if administered to individuals with loiasis; therefore, community treatment with ivermectin should be cautiously employed [35]. Additionally, ivermectin is not licensed for human use in most European countries. Hence, the drug needs to be imported through international pharmacies from, e.g., France and the Netherlands, where ivermectin is registered.

## Public Health Strategies for the Control of Strongyloidiasis in Endemic Countries

Crucially important agenda items of the meeting were related to updates on the disease burden in endemic countries, availability of ivermectin, and other control measures. In the remainder of this piece, a brief update is provided and current knowledge gaps are highlighted.

### Prevalence of Strongyloidiasis in Tropical and Subtropical Countries

Several meeting participants reported recent, mainly unpublished results of studies conducted in endemic areas of three continents: Africa (Ethiopia and Zanzibar), Asia (Cambodia and People’s Republic of China), and South America (Bolivia and Ecuador). Two issues emerged from these analyses: (i) considerable heterogeneity of prevalence data; and (ii) a large variety of diagnostic methods employed.

A survey performed in the northwestern part of Ethiopia in primary school children (*n* = 396) reported an *S*. *stercoralis* prevalence of 4% with stool microscopy utilizing the formalin-ether concentration technique, 12% with Baermann funnel technique, 13% with PCR, and 21% when considering the different methods combined. Observed co-infection with *S*. *stercoralis* and hookworm was higher than expected by chance [36].

Recent surveys carried out in Cambodia revealed *S*. *stercoralis* infection rates that were considerably higher than previously thought. Indeed, two large-scale, community-based surveys conducted in two provinces (Takeo and Preah Vihear) involving more than 5,000 individuals found village infection rates as high as 50%. Furthermore, high infection intensities were found among infected people, as determined by the number of microscopically observed larvae using the Baermann method [37,38]. A considerable number of gastrointestinal and dermatologic symptoms resolved after treatment with ivermectin, thus underscoring the considerable health impact of chronic strongyloidiasis [39]. A national survey is underway to assess the burden of *S*. *stercoralis* infection throughout Cambodia. It is important to note that in rural parts of Cambodia, access to adequate diagnosis and to ivermectin is lacking.

Recent data from the People’s Republic of China indicate that *S*. *stercoralis* may only occur in pockets of transmission. A review published in 2013 only reported 330 confirmed cases of *S*. *stercoralis* in the People’s Republic of China from 1973 to 2011 [40]. However, a small community-based study conducted in an ethnic minority region in Yunnan province, where hookworm and other soil-transmitted helminth infections are rampant, found an *S*. *stercoralis* prevalence as high as 12% based on a combination of different diagnostic approaches [11]. Nonetheless, the epidemiology of *S*. *stercoralis* is poorly understood in the People’s Republic of China, and ivermectin is currently not available for human use.

In the provinces of Cochabamba and Santa Cruz in Bolivia, among adult patients (≥18 years) at high risk of complications, the serologic and coproparasitologic prevalence was 23.0% and 7.6%, respectively. Given the known diagnostic performance of the serologic test, the actual prevalence of strongyloidiasis is estimated around 20% [41]. In Bolivia, direct fecal smear is the most commonly employed technique in clinical practice. Given the poor awareness on *S*. *stercoralis*, even among healthcare workers, more sensitive diagnostic techniques are rarely employed. Since mid-2015, ivermectin is registered in Bolivia. However, access is difficult and a concerted control strategy remains elusive.

*S*. *stercoralis* can even be at high prevalence in marginalized populations in high-income countries, but data are lacking. Aboriginal Australians living in rural communities may have prevalences greater than 15% [42,43]. Seroprevalence was 21% among 1,012 Aboriginal people from Arnhem Land, Northern Territory, decreasing to 6% 12 months after community-wide ivermectin administration [44]. Information on the epidemiology is very deficient, even in a high-income country like Australia.

### The Role of Preventive Chemotherapy with Ivermectin for the Control of Strongyloidiasis

A recently published study documented the reduction of *S*. *stercoralis* prevalence as an ancillary benefit of regular mass administration of ivermectin in Esmeraldas province in Ecuador, which had been carried out in the context of the onchocerciasis elimination program [45]. While the prevalence of *S*. *stercoralis* declined dramatically in the onchocerciasis intervention area, it remained high in the non-onchocerciasis endemic parts of the province, where no preventive chemotherapy with ivermectin took place. Similar observations have been made on the two islands of Zanzibar (Pemba and Unguja), Tanzania, where the Global Program for the Elimination of Lymphatic Filariasis (GPELF) has employed regular mass treatment with ivermectin and albendazole over several years (2001–2006). An independent assessment of the prevalence of *S*. *stercoralis* infections on Pemba Island showed a considerable reduction from 41% in 1998 to around 7% in 2013, seven years after the LF elimination program (Box 3).

Moreover, these findings confirm observational data from two cross-sectional surveys carried out in 1994 and 2006/2007 on Unguja Island that found an approximately 80% lower prevalence of *S*. *stercoralis* in the 2006/2007 survey compared to the situation in 1994 [46]. These data provide evidence that *S*. *stercoralis*-endemic communities might benefit substantially from regular ivermectin administration within the frame of LF control/elimination programs.

Collecting available data to demonstrate the impact of large-scale administration of ivermectin on the prevalence of *S*. *stercoralis* will give more insights on the effectiveness of mass drug administration and might contribute to include *S*. *stercoralis* in the WHO preventive chemotherapy strategy against soil-transmitted helminthiasis. However, preliminary data analysis shows that the interpretation of available data is frequently hampered by the paucity of studies conducted with diagnostic tests with reasonable sensitivity and by inherent differences in the design of most studies, which renders comparisons difficult. In a recent community-based study in eight villages in northern Cambodia, a cohort of 1,269 *S*. *stercoralis* patients were followed over a 2-year period after treatment with ivermectin (once yearly with a single oral dose of 200 μg/kg). The intervention proved to be highly beneficial, particularly in combination with improved sanitation; reinfection rates with *S*. *stercoralis* measured 1 year after the first or second treatment were 14.4% and 11.0%, respectively [47].

Hence, well-designed community-based cohort studies are needed to confirm the effectiveness of ivermectin treatment against *S*. *stercoralis* beyond the individual patient level. Additionally, a more accurate epidemiologic database would provide more reliable estimates on the true burden of strongyloidiasis. Taken together, such information will allow for calculating the need and the demand for ivermectin tablets to control strongyloidiasis (market analysis), which is an essential prerequisite for any drug donation program that may potentially follow. Such a market analysis should be done in concert with the scabies research and control community, which is currently looking for ivermectin donation to reduce the scourge of scabies and other ectoparasites [50,51]. Beyond strongyloidiasis, LF, and scabies, additional health benefits may accrue. There is, for instance, evidence that ivermectin-containing blood meals have a negative impact on the lifespan of *Anopheles* mosquitoes, thus reducing malaria transmission. It has been speculated that this strategy might play a role in future malaria control and elimination efforts [52]. Last, but not least, the combination of ivermectin-albendazole has recently been proposed as the first-priority combination to be implemented for the treatment, control, and elimination of soil-transmitted helminthiasis. The underlying rationale for the use of co-administered drugs is their higher efficacy, not only for *S*. *stercoralis* but also for other soil-transmitted helminths (particularly *T*. *trichiura*), than drugs being administered in mono-therapy, which in turn will reduce the risk of anthelmintic resistance against current anthelmintic drugs [53,54]. Therefore, merging the evidence of the use and demand of ivermectin for the treatment of strongyloidiasis, scabies, LF, soil-transmitted helminthiasis and, pending further research, malaria control/elimination might eventually lead to the implementation of a large ivermectin donation program. More generally speaking, improved availability of ivermectin at a global scale is warranted. In combination with a market analysis and exploration of potential public-private mechanisms, this may also stimulate generic producers and pharmaceutical companies to enter the market and help make ivermectin more widely available. However, important prerequisites have to be fulfilled; e.g., previous, cost-intensive bioequivalence studies would need to be performed to achieve pre-qualification by WHO of generic ivermectin production.

## Conclusions

The following recommendations are proposed by StrongNet, the newly instituted international network on strongyloidiasis:

1. There is a pressing need for an in-depth epidemiologic analysis to further improve the understanding of the global burden of strongyloidiasis. Research questions on the effects of larval output on morbidity and the type of gut pathology caused by *S*. *stercoralis* need to be addressed without delay.
2. Sensitive diagnostic tools should continue to be developed, with the ultimate goal of producing a highly sensitive, stool-based, POC RDT for individual patient diagnosis as well as for surveillance purposes.
3. Ivermectin should be made available for community-based treatment in *S*. *stercoralis*-endemic communities and for improved patient management, particularly in LMIC.
4. The management of strongyloidiasis in individual patients encountered in daily clinical practice and a more population-oriented approach to control and eliminate strongyloidiasis as a public health problem in endemic countries should be tackled as two separate entities.
5. Ivermectin has demonstrated a key role in the control of several NTDs and might potentially be utilized in malaria elimination campaigns. A synergistic collaboration with partners of the malaria constituency, scabies control, LF, soil-transmitted helminthiasis, and onchocerciasis control and elimination programs should be encouraged, yet care is indicated in areas where loiasis co-exists.
6. Awareness and political pressure must be enhanced and drug companies engaged to set the basis of a donation program for the distribution of ivermectin to LMIC and to lower the cost of the drug for human use. However, solutions that do not solely rely on donation must also be identified to ensure the sustainability of such programs that are readily tailored to social-ecological contexts. Appropriate advocacy tools should be used to draw the media’s attention to the general neglect of strongyloidiasis.

We feel that the time is ripe to push the agenda to facilitate the availability of ivermectin in LMIC and to add this drug to the available set of interventions for a comprehensive and integrated control of several NTDs. The major goals of the newly established StrongNet are to fill existing knowledge gaps on epidemiologic research and diagnostics, strengthen evidence, and provide advocacy for broader ivermectin availability.
